# The chain of survival and rehabilitation for sepsis: concepts and proposals for healthcare trajectory optimization

**DOI:** 10.1186/s13613-024-01282-6

**Published:** 2024-04-16

**Authors:** Romain Jouffroy, Félix Djossou, Rémi Neviere, Samir Jaber, Benoît Vivien, Nicholas Heming, Papa Gueye

**Affiliations:** 1 Intensive Care Unit, Ambroise Paré Hospital, Assistance Publique - Hôpitaux de Paris, Boulogne Billancourt, France; 2 Centre de recherche en Epidémiologie et Santé des Populations - U1018 INSERM - Paris Saclay University, Paris, France; 3 EA 7329 - Institut de Recherche Médicale et d’Épidémiologie du Sport – Institut National du Sport, de l’Expertise et de la Performance, Paris, France; 4 Service des Maladies Infectieuses et Tropicales, Guyane and Laboratoire Ecosystèmes Amazoniens et Pathologie Tropicale EA 3593, Centre Hospitalier de Cayenne, Université de Guyane, Cayenne, France; 5 Service des Explorations Fonctionnelles Centre Hospitalier Universitaire de Martinique et UR5_3 PC2E Pathologie Cardiaque, toxicité Environnementale et Envenimations (ex EA7525, Université des Antilles, Antilles, France; 6 Anesthesiology and Intensive Care; Anesthesia and Critical Care Department B, Saint Eloi Teaching Hospital, University of Montpellier, INSERM U1046, Centre Hospitalier Universitaire Montpellier, Montpellier, 34295 France; 7 Service d’Anesthésie Réanimation, SAMU de Paris, Hôpital Universitaire Necker - Enfants Malades, Assistance Publique - Hôpitaux de Paris, Université Paris Cité, Paris, France; 8 Department of Intensive Care, Raymond Poincaré Hospital, Laboratory of Infection & Inflammation - U1173, School of Medicine Simone Veil, FHU SEPSIS (Saclay and Paris Seine Nord Endeavour to PerSonalize Interventions for Sepsis), APHP University Versailles Saint Quentin - University Paris Saclay, University Versailles Saint Quentin - University Paris Saclay, INSERM, Garches, Garches, 92380 France; 9 SAMU 972, Centre Hospitalier Universitaire de Martinique, Fort-de-France Martinique, University of the Antilles, French West Indies, Antilles, France; 10 Service de Médecine Intensive Réanimation, Hôpital Universitaire Ambroise Paré, Assistance Publique – Hôpitaux de Paris, and Paris Saclay University, Saclay, France

**Keywords:** Healthcare trajectory, Network, Early therapy, sepsis, Shock

## Abstract

This article describes the structures and processes involved in healthcare delivery for sepsis, from the prehospital setting until rehabilitation. Quality improvement initiatives in sepsis may reduce both morbidity and mortality. Positive outcomes are more likely when the following steps are optimized: early recognition, severity assessment, prehospital emergency medical system activation when available, early therapy (antimicrobials and hemodynamic optimization), early orientation to an adequate facility (emergency room, operating theater or intensive care unit), in-hospital organ failure resuscitation associated with source control, and finally a comprehensive rehabilitation program. Such a trajectory of care dedicated to sepsis amounts to a chain of survival and rehabilitation for sepsis. Implementation of this chain of survival and rehabilitation for sepsis requires full interconnection between each link. To date, despite regular international recommendations updates, the adherence to sepsis guidelines remains low leading to a considerable burden of the disease. Developing and optimizing such an integrated network could significantly reduce sepsis related mortality and morbidity.

## Sepsis: a major health issue

Over 50 million patients suffer from sepsis every year [1–3]. The incidence of sepsis is approximately 300 per 100,000 inhabitants in the US [4] leading to nearly 270,000 deaths. With an incidence of 41.5 million cases per year leading to 8.2 million deaths in 2017, the burden of sepsis is highest in areas with the lowest soci-demographic index [5, 6]. Sepsis accounts for 20% of deaths worldwide [6]. The World Health organization (WHO) recently recognized sepsis as a leading public health issue. The overall burden of sepsis is increasing [7] due to a more and more aging and frailer population [8–11]. The short-term economic burden of sepsis is related to hospital costs [6, 12–14], while long-term burden is related to the support of subsequent neurocognitive, mental health sequalae or physical disabilities [8, 15, 16]. US cost-of-illness studies indicate that the direct cost of sepsis per patient is nearly 30 000$ [4]. Indirect costs are 3 to 4 times higher, arising mainly from productivity loss [4].

## Early recognition and therapy

**Fig. 1:**
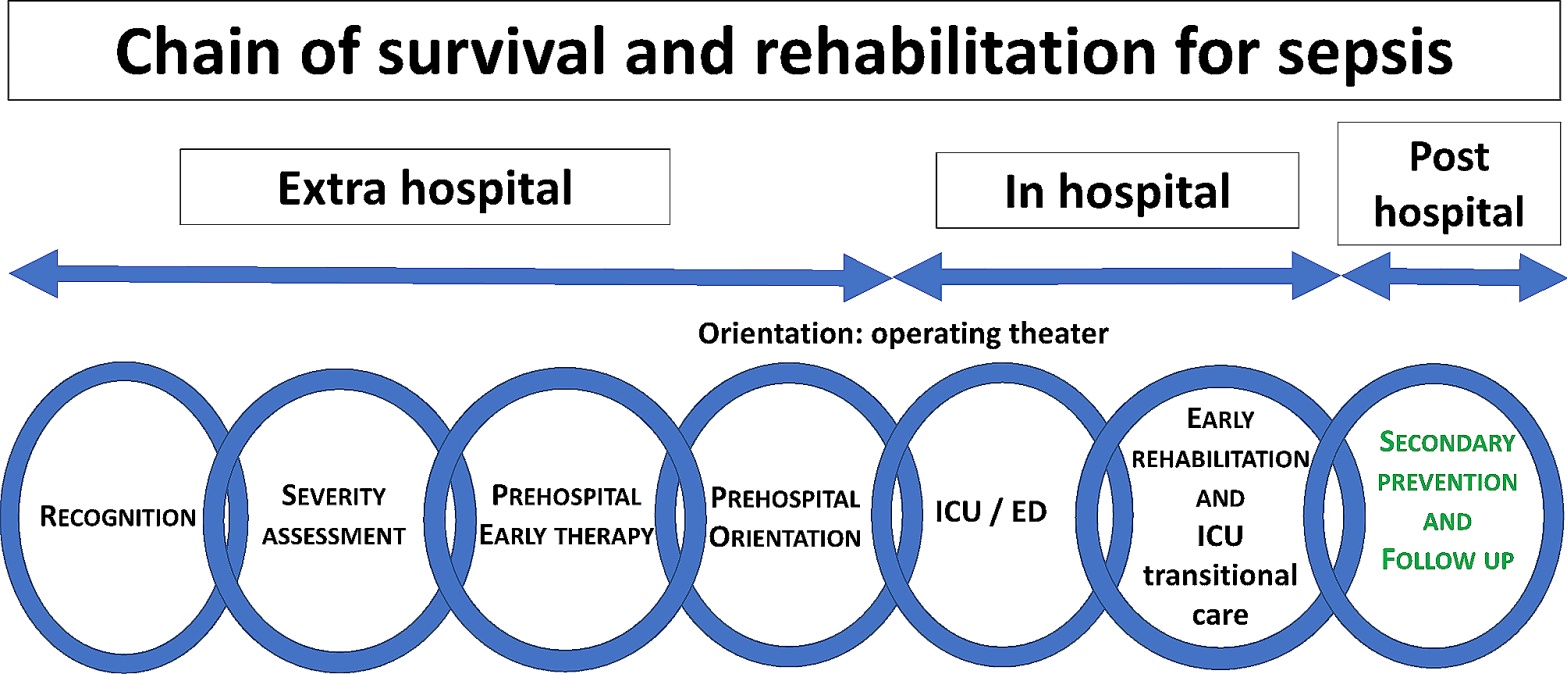
The concept of “chain of survival and rehabilitation” for sepsis ICU: Intensive Care Unit, ED: emergency department

Up to 70% of all cases of sepsis may be identified in the community [17]. Early warning is therefore essential, through the timely initiation of the specific “chain of survival and rehabilitation for sepsis” (Fig. 1) that summarizes a theoretical optimal pathway of care for patients suffering from sepsis.

The chain of survival metaphor, previously used to describe the management of cardiac arrest, captures the complexity of coordinating prehospital emergency medical service (EMS) and hospital care [18]. The concept is straightforward, since prehospital EMS and hospital-based wards are separate entities. In addition, early treatment is associated with a better outcome. However, to initiate the chain of survival, sepsis must first be properly identified. Due to a lack of specific signs and symptoms of sepsis, significant time may be lost before the identification of the condition and therefore for assistance to be requested. Pre-hospital identification of sepsis can be achieved by a witness or a relative, a primary health caregiver or a pre-hospital EMS dispatcher. The role of the prehospital EMS dispatcher, when available, is to determine the severity of the condition and to decide if mandatory to send an emergency medical team. Overall, recognition of the seriousness of the condition, calling for help, and ambulance response time (time interval from reception of the call to the arrival of the emergency medical team at the scene), all increase the delay before prehospital EMS intervention and implementation of an emergent therapy. However, in low and lower middle income countries; there may be no possibility to call for help and/or no ambulance available for hospital transportation. A solution is to improve the recognition of sepsis by relatives and primary caregivers to reduce the delay between sepsis identification and care initiation [19]. Kironji et al. reported that in low and lower-middle income countries, policy makers and researchers should focus their efforts to increase transport availability, caregivers training and access to the out-of-hospital emergency care system [19].

Educational efforts seek to raise awareness of sepsis among the public and professionals [13, 20–23]. Indeed, recent studies report an incomplete understanding of sepsis among the public [13]. Earlier initiation of the survival chain through education should help to improve patient outcome.

## Differences of organization, structure and process of prehospital emergency care for sepsis

In sepsis, patient outcomes are influenced by the process of care, primarily time intervals between the occurrence of sepsis and the delivery of four major interventions: recognition, severity assessment, early therapy, and transfer to the adequate facility [24–26]. These steps are included in “bundles of care”, as part of a general strategy recommended to improve the outcome of sepsis [27, 28]. Indeed, patient outcome is determined by the time with which these interventions are successfully delivered [28–31]. Delays in implementing these interventions are related to the organization of the healthcare system, which varies greatly from country to country, and depends on the available personnel and equipment [32]. For early therapy to be successfully delivered, the management of medical emergencies occurring in the community needs to be optimized. For example, in France, prehospital EMS is based on the SAMU (Urgent Medical Aid Service), a public organization providing a medical response to prehospital emergencies. The central component of SAMU is the dispatching center, where a team of physicians and assistants answer requests for medical assistance through a dedicated phone line [33]. SAMU also manages the SMUR (Mobile Emergency and Resuscitation Service) with mobile intensive care units (mICU), which provides advanced out-of-hospital therapy and may transport the patient. A similar organization to the French original SAMU system nowadays exists in several European countries, but also in low-income countries [24–26]. By contrast, in low and lower middle income countries, healthcare resources are scarce [34], leading to increased distance and time to access the appropriate healthcare structure (35–37). To date, no single care delivery model exists in low and lower-middle income countries because of the heterogeneity of the local context. To develop multi-faceted approaches through education, research, and policy should be considered [35].

## Prehospital recognition and severity assessment of sepsis

A recent study by Parsons Leigh et al. [13] reported incomplete awareness and understanding of sepsis among the Canadian public, confirming earlier findings [20–23]. These observations confirm the positive impact of awareness programs performed among primary care and hospital health caregivers, both physicians and nurses, paired with the dissemination of sepsis guidelines and practice bundles allowing the improvement of sepsis diagnosis and the reduction of response care delays [36, 37]. Because most cases of sepsis occur in the community [17], it makes sense to promote awareness of sepsis among the public [38, 39]. General practitioners, nurses, paramedics, prehospital EMS call centers and prehospital emergency medical teams all play a crucial role in the early identification of sepsis [30, 36, 40]. To optimize the management of sepsis, the general practitioner, e.g., often the first witness, or sometimes the nurse, must be able to recognize the severity of the condition and to alert the prehospital EMS call center in order to initiate prehospital care [36]. Consequently, general practitioners play a major role in the overall quality of care of sepsis [41]. Nevertheless, a French study conducted among a sample of general practitioners in the greater Paris area reported a lack of knowledge about sepsis and its management [42]. Severity assessment is also a major issue. A simple, easy tool to assess sepsis severity would therefore be helpful [43]. The Quick SOFA score (qSOFA), which is a simplified SOFA score, was developed and suggested for such a purpose. The qSOFA score is composed of three clinical variables: impaired consciousness, systolic blood pressure (SBP) ≤ 100mmHg and respiratory rate (RR) ≥ 22/min and allows a rapid identification of the most severe forms of sepsis [36]. However, despite its simplicity, qSOFA has a limited sensitivity and is not recommend for sepsis screening [25, 28]. Indeed, sepsis is difficult to diagnose, either at the bedside or listening to a distressed bystander/family member in a call center. Scoring systems have been developed to try to alleviate some of these issues. Unfortunately, qSOFA, MRST, MEWS and PRESEP scores do not reliably predict ICU admission [44]. A reliable score for pre-hospital triage to predict the need for ICU admission is still being sought.

## Prehospital emergency care and strategy

Evidence from in hospital studies indicate that early antibiotic therapy and hemodynamic optimization [27] improve outcomes in sepsis [36, 45–51], especially for the sicker patients [45]. Hemodynamic optimization relies on volume expansion and early norepinephrine infusion [36, 40] with a target mean blood pressure (MBP) of 65 mmHg [30, 52]. A shortened delay to correct hypotension is associated with improved outcomes [53–56]. Early antibiotic therapy administration is associated with sepsis morbidity and mortality decrease [57, 58]. Current guidelines recommend that antibiotic therapy be started within the first 3 h after sepsis recognition and diagnosis, or even as soon as possible in patients with high likelihood for sepsis [30, 36, 52]. Nevertheless, the right equilibrium, between the potential benefits versus unintended harms of antibiotic therapy [59], needs clarification in order to avoid the unwarranted administration of antibiotics to patients with non-infectious shock [60].

It is expected that an early management strategy will be more effective for the sicker and the frailest patients suffering from sepsis. Since every link in the chain of survival and rehabilitation for sepsis must be considered, we cannot rule out the impact of prehospital EMS organization on outcome [59]. For instance, a direct admission to the intensive care unit contributes to outcome improvement. If evidence-based medicine suggests the beneficial effect of the use of prehospital antibiotic therapy administration for severely ill patients [61–63], the impact of the prehospital EMS organization is not established [64].

In France, because 70% of sepsis occur in the community [17], and because prehospital care duration is nearly 60 to 90 min [45], the prehospital period offers a unique opportunity to save lives, by decreasing time-to-antibiotic therapy administration and by decreasing time-to-hemodynamic optimization [65]. Prehospital studies report a positive association between survival and early antibiotic therapy [45] and/or hemodynamic optimization [55, 66], based on early fluid expansion and/or norepinephrine administration [55, 66]. In low and lower-middle income countries, because of the scarcity of emergency care, the distance and time to access appropriate services [67–69], the development of emergency care systems is a growing focus. A recent review reported that, beyond the prehospital EMS organization, in low and lower-middle income countries, e.g. mainly in Africa, efforts should focus on improving out-of-hospital emergency care by increasing the availability of transport, caregiver training and patient access to the out-of-hospital emergency care system [19]. In these countries, the development and implementation of these three measures would allow to reduce facilities access delays, as well as allowing earlier antibiotic therapy and/or hemodynamic optimization for septic patients.

Consequently, policy makers, researcher and prehospital caregivers should be aware of their crucial role in early sepsis care [19]. Beyond antibiotic therapy administration and hemodynamic optimization, prehospital caregivers must also ensure that their patient is brought to the adequate facility for comprehensive treatment of sepsis. Controlling the source of sepsis impacts as much the outcome that early antibiotic therapy and hemodynamic optimization. As a result, prehospital caregivers have a key role on deciding in which facility the patient should be admitted. Whilst hemodynamic optimization and antibiotic therapy administration do not require any specific facility, sepsis source control may require a surgical procedure, i.e. peritonitis, which must be taken into account in the prehospital decision to refer the patient. The decision is mainly based on clinical assessment but may be helped by ultrasonography evaluation [70, 71], which is widely available in high-income countries and much less in low- and middle-income countries. Although the involvement of public health and healthcare policies is of paramount importance in determining which pre-hospital medical devices are available, the clinical evaluation remains still available.

### In hospital care: emergency department, intensive care unit and ward to rehabilitation

#### Emergency department

Because septic patients may be primarily admitted to the emergency department, prior ICU admission or due to the lack of immediately available ICU bed, the guidelines for sepsis management should also be apply [28]. However, the ED overcrowding induces an increase in delays of sepsis recognition, severity assessment and treatment initiation, associated with worse outcomes [72]. To offset this, sepsis rapid response teams were developed around the World aiming for the early recognition, diagnosis, severity assessment and treatment of patients suffering from sepsis with a positive impact on patients’ outcome [73]. The early identification based on electronic tools and/or human collaborative approach with interdisciplinary teams improves sepsis bedside huddle and bundle compliance and sepsis outcomes in the emergency department [74], allowing shortening entry in the bundle [75] and decreasing inpatient hospital mortality rates, ED length of stay and hospital length of stay [76]. The activation, composition and rules of sepsis rapid response teams must be thought out and considered on a case-by-case, depending on local resources facility and the needs of the patient to encourage bundle adherence and to hope sepsis outcome improvement [28]. Having a clinical pharmacist on sepsis rapid response teams allows the optimal selection and dosing of initial dose, reduces the time to initiation of antibiotic therapy leading to a reduced inpatient mortality [77].

#### ICU care

Recently updated guidelines summarize treatments and strategies for managing sepsis [28].

A special attention on antibiotic therapy management is therefore essential. Sepsis leads to alterations of antibiotics PK/PD parameters because of renal clearance alteration [78, 79] and/or extracorporeal supports [80], may reducing blood concentrations leading to failure, or increasing drug toxicity [81, 82], therefore, guidelines recommend optimizing dosing antibiotic therapy based on PK/PD principles and specific drug properties [28]. To avoid the development of antimicrobial resistance, a daily assessment for de-escalation of antimicrobials over using fixed durations of therapy without daily reassessment for de-escalation is recommended [28]. This strategy is associated with short-term mortality improvement [83]. Despite regularly updated recommendations, recent studies reported that despite overall awareness and the importance of early diagnosis and treatment is high among healthcare practitioner, the adherence to sepsis bundles is well below the standard of care leading to important gaps and obstacles in reaching optimal care both in adults and pediatrics [84–86], reinforcing the importance of implementing a specific pathway of sepsis care.

The COVID 19 pandemic revealed to the World the inequity of access to an adequately equipped and staffed ICU bed because of the lack of ICU beds [87]. Beyond COVID 19 pandemic, the lack of ICU beds is a daily problem even more in low-income countries where most ICUs are located in large referral hospitals [88] leading to issues for the management of sepsis.

Bundles of care also aim at reducing the adverse effects of critical illness to optimize patient recovery and outcomes [89]. Recent studies report that early rehabilitation, e.g., started within 3 days of ICU admission, was associated with decreased length of stay and improved daily activities after hospital discharge [90, 91], indicating the importance of the early rehabilitation within the ICU. For this purpose, since 2013, the American College of Critical Care Medicine, the Society of Critical Care Medicine and the American Society of Health-System Pharmacists, updated the in-ICU PAD (Pain, Agitation, and Delirium) guidelines to improve critically ill patient management [92]. More recently, the ABCDEF bundle, including many elements of the in-ICU PAD guidelines, was proposed. Briefly, the ABCDEF bundle includes: Assess, Prevent, and Manage Pain (A), Both Spontaneous Awakening Trials (SAT) and Spontaneous Breathing Trials (SBT) (B), Choice of analgesia and sedation (C), Delirium: Assess, Prevent, and Manage (D), Early mobility and Exercise (E), and Family engagement and empowerment (F) in order to early optimize resources utilization [89].

#### Post ICU care

Among survivors, nearly 50% recover, 30% die within the first year, and 15% suffer from severe persistent impairments [93]. The “post-sepsis syndrome (PSS)” associates physical, medical, cognitive, and mental health sequalae, responsible of long-term morbidity [16]. Prior to PSS, the post-intensive care syndrome (PICS) involves physical, cognitive, and mental impairments occurring during ICU stay or after ICU/hospital discharge, impairing the long-term outcome of survivors [94–98]. In order to combat PSS and PICS, post discharge rehabilitation strategies are effective and associated with a reduced risk of 10-year mortality in sepsis survivors [99]. Surviving Sepsis Campaign guidelines have a particular focus on continuing rehabilitation to improve functional outcomes during and after ICU discharge [28].

Sepsis is an entity for which the evidence of post-acute care on long-term outcome is supported by evidence-based medicine [99] despite changes over time [9]. The transition point from the ICU to ward is an important stage in the patient medical history. Indeed, it is essential to prepare the transfer to the general ward accurately and correctly to avoid the risk of patient ICU-readmission associated with stress for both patients and relatives [100–104]. ICU transitional care corresponds to the care provided before, during, and after the transfer from ICU to a ward with a minimal disruption and maintaining the optimal care for the patient [105]. To ensure ICU transitional care the discharge procedure need to be safe and structured involving a multidiscipline approach [105] because it corresponds to a period of high vulnerability [106]. To date, post-acute care resources are insufficient to address the needs of sepsis survivors [93] reflected by high rates of adverse outcomes after hospital discharge from high rates of healthcare utilization to hospital readmission and increased mortality [107–109]. Best-practice guidelines were developed to guide delivery of post-acute care [93] but suffer from a gap in understanding how to best integrate interventions into the complex post-discharge setting [110, 111].

### Maximizing survival rate

The following table summarizes the differences between current practices and optimized practices according to the “chain of survival for sepsis” concept and proposals to achieve its goals (Table 1).

**Table 1:** Current and optimized practices according to the “chain of survival for sepsis” concept and proposals to achieve its goals

Current practices	Optimized practices	Proposals
Incomplete awareness and understanding	- Earlier sepsis recognition - Earlier prehospital EMS management (call and ambulance dispatch to the scene)	Educational and public service courses to raise awareness of sepsis among primary care, general practitioners, nurses, paramedics, prehospital EMS regulation call centers and prehospital emergency medical teams
Scores (qSOFA, MRST, MEWS, SIRS, NEWS, PRESEP) insufficient to predict ICU admission	Sensitive and specific scoring tools to assess sepsis severity and triage optimization	Development of a reliable score for triage and severity assessment
Wide heterogeneity of prehospital sepsis care	- Consider early antibiotic therapy administration within 3 h (or even as soon as possible in patients with high likelihood for sepsis) after sepsis recognition - Consider early hemodynamic optimization with prehospital mean blood pressure target of 65mmHg - Consider early dispatching a primary health caregiver to the scene to deliver immediate care if ambulance is not available	- Improve spatial census of public hospital services, transports availability, caregivers training and patient access to the out-of-hospital emergency care system - Promote early antibiotic therapy available in ambulance and prehospital EMS team vehicle for community (3rd generation cephalosporin) and nosocomial (piperacillin-tazobactam) respiratory, urinary, and digestive infections - Prehospital crystalloids fluid expansion based on dynamic hemodynamic parameters - Promote early prehospital norepinephrine administration to reach mean blood pressure target of 65mmHg - Promote early primary health caregiver dispatching to the scene to deliver care
Admission to the emergency department or ICU admission	Immediate ICU admission or immediate life-saving emergency room in the ED admission or life-threatening emergency room admission	Educational and courses for prehospital EMS regulation call centers, prehospital emergency medical teams and ICU to facilitate admission
Delays of sepsis recognition, severity assessment and treatment initiation due to ED overcrowding	Reduction of delays for sepsis recognition, severity assessment and treatment initiation in the ED	Promote sepsis rapid response teams development
Delayed ICU admission due to a lack of beds	Adequate number of ICU beds	Promote public health and healthcare policies involvement to increase the number of ICU beds
Low adherence to sepsis bundles	Maximising sepsis bundles adherence	Initial and refresher educational and courses for healthcare practitioners
Rehabilitation practices variations between ICUs	Early rehabilitation within 3 days of ICU admission	Educational courses for ICU teams to initiate early rehabilitation
Insufficient post-acute care resources	Agreement with best-practice guidelines	Improve issues understanding to better integrate interventions into the complex post-discharge setting

Future studies should determine the impact of implementing an optimized care pathway for sepsis. Without a proper and coordinated implementation of the “chain of survival and rehabilitation for sepsis”, such objectives will not be achieved.

## Conclusion

Early access to the “chain of survival and rehabilitation for sepsis” ensures the early initiation of life saving treatments followed by the orientation of the patient to the adequate facility for advanced care. Earlier warning will be ensured by raising awareness of the condition among general practitioners, nurses, paramedics, prehospital caregivers and the general public. Earlier advanced care, based mainly on early antibiotic therapy and hemodynamic optimization, is possible independently of the prehospital emergency medical service organization even for primary health care when no ambulance can be dispatch to the scene. Triaging and admission to the adequate facility are essential for adequate source control. Advanced in hospital care helps overcome organ failure while waiting for the cause of sepsis to be treated. Rehabilitation is essential for survivors to recover an acceptable quality of life.

The ongoing public health challenge appears to be the development of coordinated actions, starting at the prehospital setting right through to rehabilitation, to be delivered as quickly as possible, thereby enhancing successful recovery for patients suffering from sepsis.

## Data Availability

Not applicable.
